# Nonadiabatic Derivative Couplings Calculated Using Information of Potential Energy Surfaces without Wavefunctions: Ab Initio and Machine Learning Implementations

**DOI:** 10.3390/molecules28104222

**Published:** 2023-05-21

**Authors:** Wen-Kai Chen, Sheng-Rui Wang, Xiang-Yang Liu, Wei-Hai Fang, Ganglong Cui

**Affiliations:** 1 Hebei Key Laboratory of Inorganic Nano-Materials, College of Chemistry and Materials Science, Hebei Normal University, Shijiazhuang 050024, China; 2 Key Laboratory of Theoretical and Computational Photochemistry, Ministry of Education, College of Chemistry, Beijing Normal University, Beijing 100875, China; 3 Hefei National Laboratory, Hefei 230088, China; 4 College of Chemistry and Material Science, Sichuan Normal University, Chengdu 610068, China

**Keywords:** nonadiabatic couplings, machine learning, excited states

## Abstract

In this work, we implemented an approximate algorithm for calculating nonadiabatic coupling matrix elements (NACMEs) of a polyatomic system with ab initio methods and machine learning (ML) models. Utilizing this algorithm, one can calculate NACMEs using only the information of potential energy surfaces (PESs), i.e., energies, and gradients as well as Hessian matrix elements. We used a realistic system, namely CH_2_NH, to compare NACMEs calculated by this approximate PES-based algorithm and the accurate wavefunction-based algorithm. Our results show that this approximate PES-based algorithm can give very accurate results comparable to the wavefunction-based algorithm except at energetically degenerate points, i.e., conical intersections. We also tested a machine learning (ML)-trained model with this approximate PES-based algorithm, which also supplied similarly accurate NACMEs but more efficiently. The advantage of this PES-based algorithm is its significant potential to combine with electronic structure methods that do not implement wavefunction-based algorithms, low-scaling energy-based fragment methods, etc., and in particular efficient ML models, to compute NACMEs. The present work could encourage further research on nonadiabatic processes of large systems simulated by ab initio nonadiabatic dynamics simulation methods in which NACMEs are always required.

## 1. Introduction

The Born–Oppenheimer (B-O) approximation is widely used in the research of computational and theoretical chemistry when potential energy surfaces (PESs) are far away from each other. However, the B-O approximation will break down when two or more PESs come close or even cross. In such a situation, nonadiabatic effects are non-negligible and nonadiabatic couplings between different PESs should be considered seriously. Nonadiabatic coupling matrix elements (NACMEs) are important physical quantities that are used for measuring coupling strengths between different adiabatic electronic states. Additionally, NACMEs play important roles in calculating non-radiative rates and nonadiabatic molecular dynamic (NAMD) simulations [1,2,3,4,5,6,7,8,9,10]. However, the computational cost of NACMEs is generally pretty expensive using ab initio methods. Moreover, not all electronic structure programs and methods have implemented wavefunction-based algorithms for calculating NACMEs yet. From this point of view, it is desirable to develop a simple and generalized method for obtaining NACMEs which can be easily adopted by most excited-state electronic structure methods. To achieve this target, Köppel et al. proposed a strategy for constructing diabatic states from PESs of adiabatic states, which can compute nonadiabatic couplings without wavefunction information [11]. Lasorne et al. re-investigated and compared wavefunction- and PES-based NACMEs and suggested that PES-based NACMEs are accurate in regions around conical intersections [12]. Richardson reported a new machine learning (ML) approach for eliminating the issue with the double-valued nature of NACMEs by a set of auxiliary single-valued functions [13]. Recently, Baeck and An developed a practical approximation for the calculation of NACMEs and successfully applied it in one-dimensional model systems [14,15]. This method is easy to implement and has the potential to combine with electronic structure methods that can provide PES information. Very recently, Gastegger and coworkers used this approximate PES-based algorithm in their developed SchNarc machine learning approach to calculate NACMEs for NAMD simulations [16]. However, the accuracy of this approximate method to calculate NACMEs for polyatomic molecular systems still needs comprehensive benchmarks and in-depth analysis.

**Figure 1:**
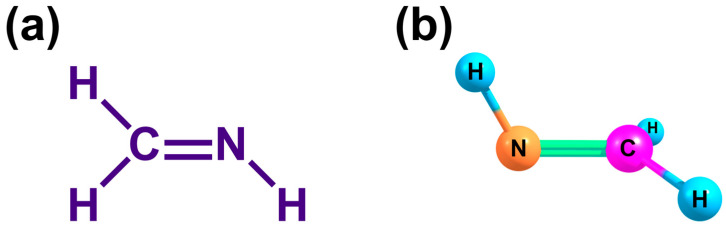
A polyatomic system used in this work. (**a**) CH_2_NH molecule; (**b**) structure of the optimized conical intersection (CI) of CH_2_NH.

Motivated by these facts, we have in this work implemented an approximate PES-based method for NACMEs using a realistic system CH_2_NH (Figure 1) and proved that this algorithm can give accurate NACMEs if PES information (i.e., energies, gradients and Hessian matrices) can be accurately provided by either ab initio methods or ML-trained models. Our work indicates that this approximate method performs very well even near PES regions with quasi-degenerate energies. However, this approximate algorithm will fail and become divergent if relevant PESs are rigorously degenerate in energy, i.e., truly conical intersections (CIs). Nevertheless, this PES-based algorithm remains useful and beneficial for combining electronic structure methods, energy-based fragment methods, and ML models, which can provide PES information but with either difficult- or impossible-to-supply wavefunctions, and has potential applications in nonadiabatic dynamics simulations, etc.

## 2. Results and Discussion

### 2.1. Comparison of Wavefunction- and PES-Based NACMEs 

**Figure 2:**
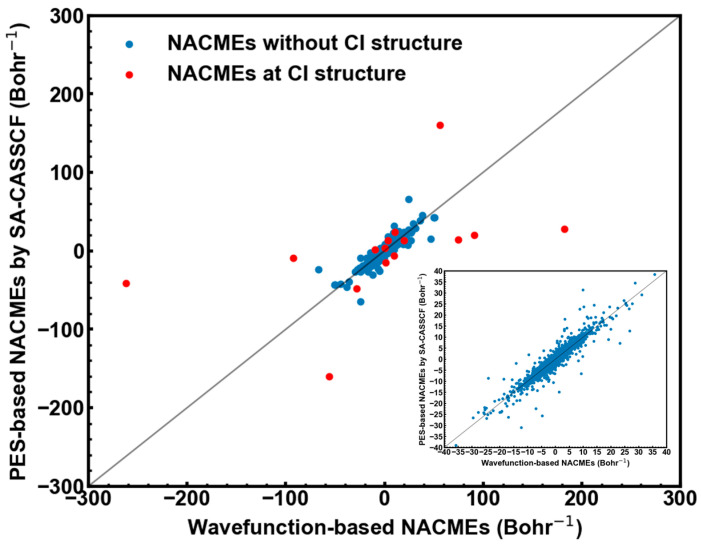
Comparison of wavefunction- and PES-based NACMEs at the CI structure (red points) and at the structures far away from the CI structure (blue points). Insert panel: comparison of NACMEs at the structures far away from the CI structure.

Figure 2 shows the wavefunction- and PES-based NACMEs with structures near the optimized CI structure of CH_2_NH. It can be found that the PES-based NACMEs at the optimized CI structure (energy gap less than 0.17 kcal/mol) not only have large values but also are far away from the wavefunction-based NACMEs (red points in Figure 2). This is originated from the fact that NACMEs at truly degenerate CI points become divergent because their values are inversely proportional to related energy gaps. Note that at the optimized CI structure of CH_2_NH, the energy gap is only 0.17 kcal/mol, which makes NACMEs a little divergent. On the other hand, the PES-based NACMEs at the structures far away from the CI structure (energy gap larger than 0.17 kcal/mol) are very close to the wavefunction-based NACMEs (see blue points and insert panel of Figure 2). The small deviations may be caused by numerically calculated Hessian matrix elements. In total, the PES-based NACMEs become much better compared with the wavefunction-based NACMEs when the energy gap is increased.

To give insight into the accuracy of the PES-based NACMEs, we separated the structures near the optimized CI structure into several groups according to their energy gaps $\Delta E_{S_{0}S_{1}}$ (i.e., 0.17–1 kcal/mol, 1–3 kcal/mol, 3–5 kcal/mol, 5–10 kcal/mol, 10–15 kcal/mol and >15 kcal/mol). Then, the average norms (see definition in Section 3.3) of the wavefunction- and PES-based NACMEs in each group were calculated (see Table 1). Firstly, the average norm gets smaller when the energy gap $\Delta E_{S_{0}S_{1}}$ is increased, which is consistent with the discussion above(see supplementary materials). In addition, as mentioned above, the approximate PES-based algorithm fails to give good enough norm of NACMEs at the optimized CI (363.4 Bohr^−1^ vs. 240.9 Bohr^−1^). As to the other groups, the deviations of the average norms between the wavefunction- and PES-based NACMEs becomes much smaller with the increasing energy gap. For instance, the average norms of the wavefunction- and PES-based NACMEs were estimated to be 78.2 and 80.4 Bohr^−1^ when the energy gap between S_0_ and S_1_ was within 0.17–1 kcal/mol. The relative deviations for the other groups, i.e., 0.17–1 kcal/mol, 1–3 kcal/mol, 3–5 kcal/mol, 5–10 kcal/mol, 10–15 kcal/mol and > 15 kcal/mol, were calculated to be 2.8%, 3.6%, 3.8%, 4.0%, 3.6%, and 3.4%, respectively (see the last column in Table 1).

**Figure 3:**
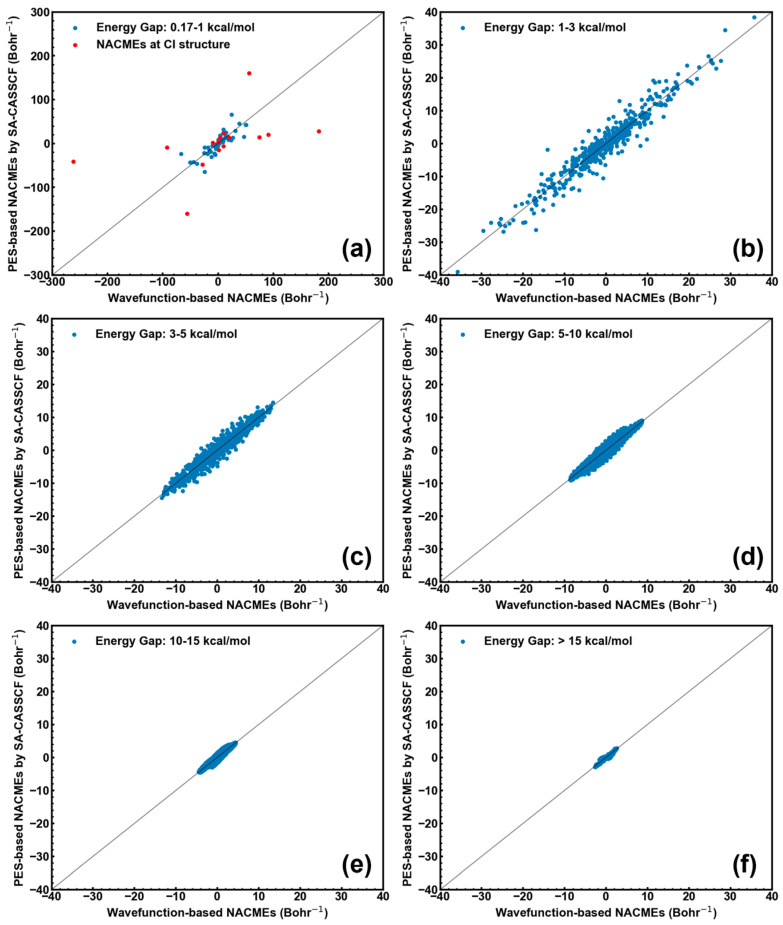
Comparison of wavefunction- and PES-based NACMEs at the optimized CI structure of CH_2_NH (red points in panel (**a**); $\Delta E_{S_{0}S_{1}}=0.17$ kcal/mol) and at the structure far away from the CI one (blue points; panels (**b**–**f**)). (**a**) 0.17–1 kcal/mol; (**b**) 1–3 kcal/mol; (**c**) 3–5 kcal/mol; (**d**) 5–10 kcal/mol; (**e**) 10–15 kcal/mol; and (**f**) >15 kcal/mol.

In order to give more in-depth comparison between the wavefunction- and PES-based NACMEs, the scattering plots for each group with the different $\Delta E_{S_{0}S_{1}}$ values (including all x/y/z components of each atom) are given in Figure 3. It is obvious that: (1) the PES-based NACMEs at the optimized CI have large deviations (red points in Figure 3a); and (2) the PES-based NACMEs are close to the wavefunction-based NACMEs for the structures far away from the optimized CI structure (see Figure 3a–f). In one sentence, these data demonstrate that the PES-based algorithm can give as accurate NACMEs as the wavefunction-based algorithm. Finally, one should also note that NACMEs calculated by both the wavefunction- and PES-based algorithms will become divergent at truly degenerate CIs. So, one can see a large deviation between both the wavefunction- and PES-based NACMEs at the optimized CI structure (energy gap less than 0.17 kcal/mol; see above).

### 2.2. PES-Based NACMEs with ML Models 

Although the PES-based NACMEs give good enough results compared with the wavefunction-based NACMEs, this PES-based algorithm remains expensive because it needs full Hessian matrix elements, which still cannot be analytically calculated for many electronic structure methods and packages nowadays, to our best knowledge. Fortunately, this shortcoming can be avoided by using trained ML models, which can calculate Hessian matrix elements analytically and efficiently, as seen in our recent papers [17]. Thus, we next used a machine learning technique, i.e., the embedding atom neural network (EANN) method [18,19,20], as an alternative to calculate the relevant information of PESs, i.e., energies, forces, Hessian matrix elements, etc.

**Figure 4:**
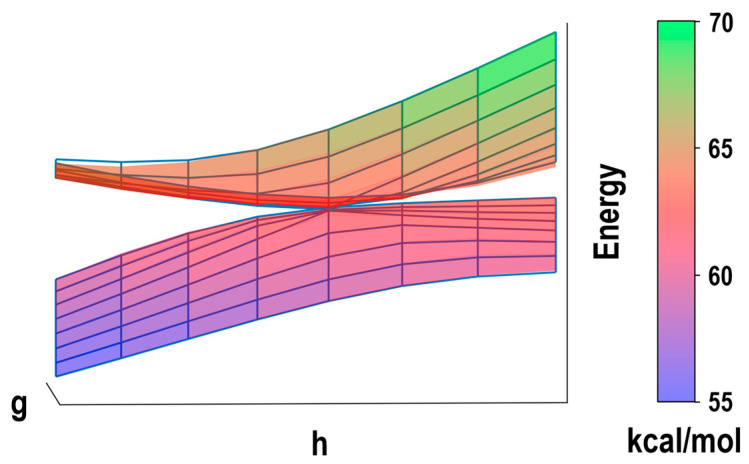
Three-dimensional PESs with respect to the two branching space vectors g and h near the S_0_/S_1_ conical intersection of CH_2_NH calculated by the SA-CASSCF method (blue grids) and the ML model trained by the EANN method (colored surfaces).

It should be noted that the ML models trained by the EANN method can provide analytical Hessian matrix elements while this ML method does not need ab initio Hessian matrix elements as input in the training stage [17,19]. The trained ML model reproduces the PESs near the optimized CI structure, calculated with the SA-CASSCF method (Figure 4), very well, which demonstrates that the trained ML model is accurate enough and can be used for the PES-based algorithm to calculate NACMEs. In fact, the previous works have proved that the ML model can give as accurate energies, forces, and Hessian matrix elements as the ab initio method as long as a sufficient quantity and quality data are provided for the ML training [16,17,21].

Similarly, we first compare the average norms of the PES-based NACMEs calculated by the ab initio (SA-CASSCF) method and the trained ML model (Table 2). In total, the deviation between the ab initio and ML-calculated NACMEs (PES-based algorithm) is very small when the energy gap is greater than 1 kcal/mol except at the optimized CI structure with an energy gap of 0.17 kcal/mol. In detail, the deviation of the average norms at the optimized CI structure is as large as 73.9% (SA-CASSCF vs. ML: 240.9 Bohr^−1^ vs. 62.9 Bohr^−1^). Moreover, the PES-based NACMEs with the ML model do not perform very well when the energy gap is in the range of 0.17–1 kcal/mol, which gives a deviation of 20.2%. Nevertheless, when the energy gap is larger than 1 kcal/mol, the deviations of the average norms become much smaller. Specifically, the deviations of average norms related to the energy gaps of 0.17–1 kcal/mol, 1–3 kcal/mol, 3–5 kcal/mol, 5–10 kcal/mol, 10–15 kcal/mol and >15 kcal/mol are estimated to be 3.6%, 9.1%, 0.6%, 2.3% and 2.7%, respectively. In terms of the above results, one can see that the PES-based NACMEs calculated by the ML model can also give accurate results, except at certain structures with extremely small energy gaps (i.e., around 1 kcal/mol).

**Figure 5:**
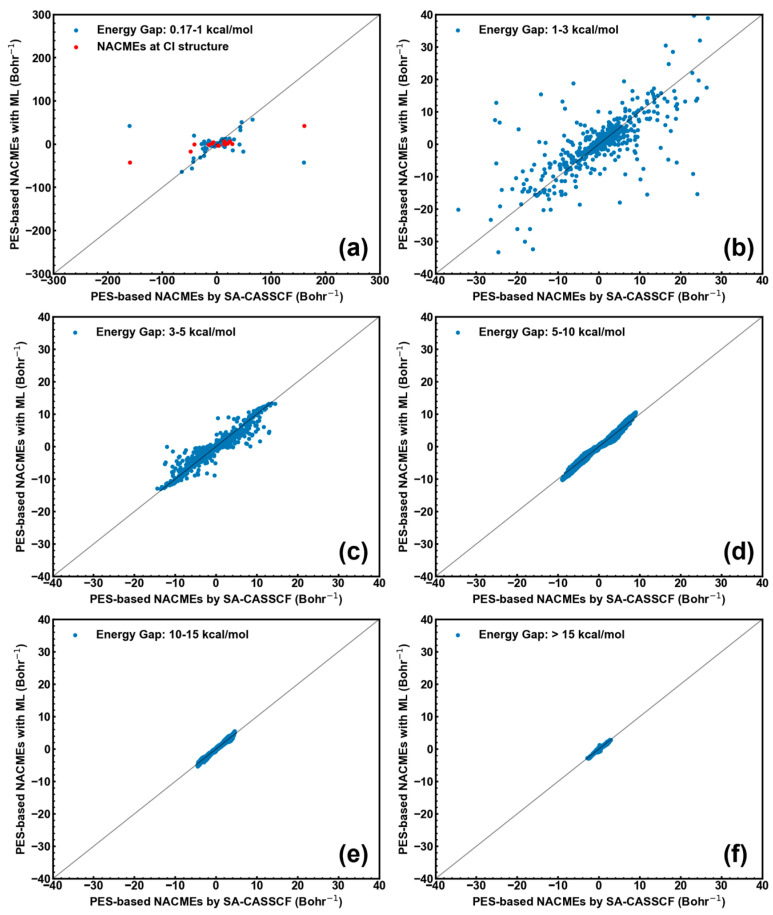
Comparison of SA-CASSCF- and ML-calculated NACMEs (PES-based algorithm) at the optimized CI structure of CH_2_NH (red points in panel (**a**); $\Delta E_{S_{0}S_{1}}=0.17$ kcal/mol) and at the structure far away from the CI one (blue points; panels (**b**–**f**)). (**a**) 0.17–1 kcal/mol; (**b**) 1–3 kcal/mol; (**c**) 3–5 kcal/mol; (**d**) 5–10 kcal/mol; (**e**) 10–15 kcal/mol; and (**f**) >15 kcal/mol.

In addition, Figure 5 compares the SA-CASSCF- and ML-calculated NACMEs (PES-based algorithm). Figure 5a shows that the ML-calculated NACMEs are to some extent far from the SA-CASSCF-calculated NACMEs at the optimized CI structure. This can be understood very well when considering that PESs are discontinuous at these energetically degenerate points (i.e., singularities). In addition, the accurate fitting of PESs at CI points is also extremely difficult for ML techniques due to this discontinuous character. Although the ML-calculated NACMEs are not so accurate at the optimized CI structure, they can give fairly good results for the structures where the energy gap is larger than 3 kcal/mol (Figure 5c–f). In summary, ML-calculated NACMEs provide reasonably accurate results except at truly degenerate points.

Finally, the computational cost of both the wavefunction- and PES-based NACMEs is discussed. As we have mentioned, the calculation of Hessian matrix elements at ab initio level is very expensive, which makes the computational cost of PES-based NACMEs high using ab initio methods. However, the combination of efficient ML models with the PES-based algorithm brings advantages because the calculation of Hessian matrix elements is cheap using ML models.

## 3. Methods

### 3.1. The Approximate PES-Based Algorithm for NACMEs

Here, we briefly introduce the approximate algorithm for NACMEs proposed by Baeck and An [14,15]. Diabatic states could be transformed from adiabatic states by a unitary transformation with an adiabatic-to-diabatic (ADT) mixing angle θ [22]:

(1) $$\left(\begin{array}{c} \Psi_{i}\\ \Psi_{j} \end{array}\right)=\left(\begin{array}{cc} \cos\theta & -\sin\theta \\ \sin\theta & \cos\theta \end{array}\right)\left(\begin{array}{c} \Phi_{i}\\ \Phi_{j} \end{array}\right)$$

where $\Psi_{i}\left(\Psi_{j}\right)$ represent diabatic states while $\Phi_{i}\left(\Phi_{j}\right)$ represent adiabatic states, respectively. The index i and j represent the indexes of different electronic states. Meanwhile, the relationship between diabatic potentials $V_{i}\left(V_{j}\right)$ and adiabatic potentials $E_{i}\left(E_{j}\right)$ can be written as follows [11]:

(2) $$\left(\begin{array}{cc} V_{i} & 0\\ 0 & V_{j} \end{array}\right)=\frac{\left(E_{i}+E_{j}\right)}{2}\left(\begin{array}{cc} 1 & 0\\ 0 & 1 \end{array}\right)+\frac{\left(E_{i}-E_{j}\right)}{2}\left(\begin{array}{cc} \cos2\theta & \sin2\theta \\ \sin2\theta & -\cos2\theta \end{array}\right).$$

Additionally, θ can be expressed by

(3) $$\theta \left(Q\right)=\theta \left(Q_{0}\right)+\int_{Q_{0}}^{Q}\langle \Phi_{j}^{a}|\partial /\partial Q^{\prime }{|\Phi }_{i}^{a}\rangle dQ^{\prime }=\theta \left(Q_{0}\right)-\int_{Q_{0}}^{Q}d_{ij}\left(Q^{\prime }\right)dQ^{\prime }$$

in which $Q_{0}$ is a reference coordinate and $d_{ij}\left(Q\right)=\langle \Phi_{j}^{a}|\partial /\partial Q{|\Phi }_{i}^{a}\rangle$ is a nonadiabatic coupling term. On the other hand, in 1981, Werner and Meyer proposed a Lorentz function $d_{ij}^{Lo}\left(Q\right)$ to approximate $d_{ij}\left(Q\right)$ as [23]

(4) $$d_{ij}^{Lo}\left(Q\right)=\frac{1}{2}\frac{\alpha }{(1+{\left[\alpha \cdot \left(Q^{\prime }-Q_{c}\right)\right]}^{2}}$$

in which $Q^{\prime }$ are nuclear coordinates of the current structure while $Q_{c}$ are coordinates of the crossing point. When $Q^{\prime }=Q_{c}$, one can obtain $\alpha =2\cdot d_{ij}^{Lo}\left(Q\right)$. However, this approximate method was not developed further until the work by Baeck and An in 2017, [15] who showed that the approximate NACMEs $d_{ij}^{appr}$ can be obtained by

(5) $$d_{ij}^{appr}=\frac{1}{2}\sqrt{\frac{\partial^{2}{\left(\Delta E_{ij}\right)}^{2}/\partial^{2}R}{\Delta E_{ij}}}$$

in which $\Delta E_{ij}$ is the energy gap between electronic states i and j, R represents the coordinates of the atoms. In addition, a more practical formula was adopted by Lasorne et al. and Westermayr et al. [12,16], which is shown below:

(6) $$d_{ij}^{appr}⊗d_{ij}^{appr}\approx \frac{\partial^{2}{\left(\Delta E_{ij}\right)}^{2}}{\partial^{2}R}-\frac{\partial \Delta E_{ij}}{2\partial R}⊗\frac{\partial \Delta E_{ij}}{2\partial R}$$

in which ⊗ means a tensor product. The final approximate PES-based NACMEs are calculated via

(7) $$d_{ij}^{appr}=v_{ij}\cdot \frac{\sqrt{\lambda_{ij}}}{\Delta E_{ij}}$$

in which $v_{ij}$ and $\lambda_{ij}$ are eigenvectors and eigenvalues of the right side of Equation (6) calculated by the singular value decomposition (SVD). Additionally, the signs (i.e., positive or negative) of the PES-based NACMEs are determined by Equation (7) directly.

### 3.2. The Embedding Atom Neural Network (EANN) Method

Nowadays, machine learning techniques are widely used in the research fields of physics, chemistry, biology, and materials [24,25,26,27,28,29,30,31,32,33,34]. In particular, using ML methods for improving computational efficiency is one of the most popular fields [21,35,36,37,38,39,40,41,42,43]. In this work, the EANN method proposed by Jiang et al. was adopted for training potential energy surfaces of polyatomic molecules because of its available Hessian matrices [20]. In the EANN method, the total energy of a system is written as the sum of all atomic energies $E_{i}$:

(8) $$E_{EANN}=\overset{N_{atom}}{\underset{i}{\sum }}E_{i}=\overset{N_{atom}}{\underset{i}{\sum }}NN_{i}\left(\rho^{i}\right)$$

in which $N_{atom}$ is the total number of atoms; $NN_{i}$ is the atomic neural network and $\rho^{i}$ is the function of the embedding electron density of the atom i, which is given by the superposition of electron densities of the surrounding atoms.

A set of Gaussian-type orbitals centered at each atom is used for calculating the embedding electron density associated with each atom:

(9) $$\Psi_{l_{x}l_{y}l_{z}}^{\alpha ,r_{s}}=x^{l_{x}}y^{l_{y}}z^{l_{z}}\exp\left(-\alpha {\left|r-r_{s}\right|}^{2}\right)$$

in which x, y and z are coordinate components of an electron coordinate vector r, while |r| is the norm of r. α and $r_{s}$ are parameters for determining the radial distribution of the atomic orbitals Ψ, and $l_{x},l_{y},l_{z}$ are indicating the orbital angular momentum components with the relationship $L=l_{x}+l_{y}+l_{z}$. With the definition of these Gaussian-type orbitals, the embedding electron density of the atom i can be calculated as the square of all the linearly combined atomic orbitals from the neighboring atoms. Then, the individual electron density from Gaussian-type orbitals is written as

(10) $$\rho_{L,\alpha ,r_{s}}^{i}=\overset{L=l_{x}+l_{y}+l_{z}}{\underset{l_{x},l_{y},l_{z}}{\sum }}\frac{L!}{l_{x}!l_{y}!l_{z}!}\left(\overset{N_{neighbor}}{\underset{j=1}{\sum }}c_{j}\Psi_{l_{x}l_{y}l_{z}}^{\alpha ,r_{s}}\left(r_{ij}\right)\right)$$

in which $r_{ij}$ is the distance between the atoms i and j, $N_{neighbor}$ is the number of the neighboring atoms of the atom i within the sphere with the radius equaling the cutoff radius $r_{c}$, and $c_{j}$ is the expansion coefficient of the Gaussian-type orbitals. Finally, the energy for each atom can be obtained from the atomic neural networks by taking ρ as its input vectors. Moreover, in order to decay the interactions between the central atom with neighbor atoms to zero smoothly, a cutoff function $f_{c}\left(r\right)$ is used in the EANN method:

(11) $$f_{c}\left(r_{ij}\right)=\{\begin{array}{cc} 0.5\left[\cos\left(\frac{\pi r_{ij}}{r_{c}}\right)+1\right], & r_{ij}<r_{c}\\ 0, & r_{ij}>r_{c} \end{array}$$

in which $r_{c}$ is the cutoff radius. Note that the same elements share the same atomic neural network. After obtaining the input vectors of the EANN method, the atomic neural network models are trained via minimizing the loss function δ(w,b) by the extreme learning machine (ELM) and Levenberg–Marquardt (LM) algorithm (ELM-LM) developed by Jiang et al. [44]. In addition, to improve the efficiency of the ML model training processes and reduce the size of the training data set, the deviations between ML-predicted and SA-CASSCF-calculated atomic forces are added into δ(w,b). Thus, the loss function δ(w,b) is expressed as follows:

(12) $$\delta \left(w,b\right)=\sum_{a=1}^{N_{data}}\left[{\left(E_{a}^{EANN}-E_{a}^{REF}\right)}^{2}+\eta \sum_{b=1}^{N_{atom}}\;\sum_{c=x,y,z}{\left(F_{a,b,c}^{EANN}-F_{a,b,c}^{REF}\right)}^{2}\right]/N_{data}$$

in which w and b are the weight and bias parameters of atomic neural network models, and $N_{data}$ is the size of the training data set. η is a parameter to control the importance of deviations of atomic forces during the training process. $E_{a}^{EANN}$ and $E_{a}^{REF}$ are energies of the ath configuration in the training data set calculated by EANN models and the ab initio method, while $F_{a,b,c}^{EANN}$ and $F_{a,b,c}^{REF}$ are the forces of the bth atom in c direction for the ath configuration, evaluated by EANN models and the ab initio method, respectively. The accuracy and efficiency of the EANN method for both isolated and periodic systems have been demonstrated in previous works [18,19,20].

### 3.3. Definition of the Average Norm of Matrices

In this work, the average norms of NAC matrices are calculated. The norm of a matrix $\left|A_{i}\right|$ is calculated as follows:

(13) $$\left|A_{i}\right|=\sqrt{\underset{n,m}{\sum }A_{i,nm}^{2}}$$

in which $A_{i,nm}$ is the matrix element of $A_{i}$. The average norm is calculated by dividing the sum of the norms of $A_{i}$ (I = 1 − n)

(14) $$\langle |A_{i}|\rangle =\frac{\sum_{i=1}^{n}\left|A_{i}\right|}{n}$$

### 3.4. Computational Details

In this work, a CH_2_NH molecule is used for demonstrating the accuracy of the approximate PES-based algorithm of NACMEs. To make direct comparisons, the wavefunction-based NACMEs of CH_2_NH are also calculated at the same SA-CASSCF level. The approximate PES-based NACMEs are calculated using the PES information (energies, gradients, and Hessian matrices) provided by the SA-CASSCF method or the trained ML model, respectively. In this work, we only consider NACMEs between S_0_ and S_1_ of CH_2_NH. An active space including two electrons and two orbitals, as well as 6-31G* basis sets, are adopted for all the SA-CASSCF calculations. All the SA-CASSCF calculations are performed with OpenMolcas-v18.09 [45,46]. The S_1_/S_0_ conical intersection structure of CH_2_NH is optimized by Gaussian16 [47].

In ML model training processes using the EANN method, the atomic neural networks with two hidden layers of 40 and 50 nodes are adopted as the atomic neural network models for C, H, and N elements. The cutoff radius $r_{c}$ is set to 9 Å, which is large enough to take all other atoms as the neighbor atoms of the center atom. The maximum orbital angular momentum L is set to 2, which is suitable for training models of PESs of CH_2_NH. On the other hand, β=0.2 and $\Delta r_{s}=1\;Å$ leads to $\alpha =\frac{\beta }{{\left(\Delta r_{s}\right)}^{2}}=0.2\;{Å}^{-2}$, which are used for determining the Gaussian-type-orbital input vectors in Equation (10) for all atomic neural networks in this work. In addition, η is set to 1 during the model training processes for balancing the importance of deviations of energies and atomic forces in the present work. More details of data collection and training process for the ML models can be found in our previous works [17,21,48].

The purpose of this work is to prove the accuracy and efficiency of the approximate PES-based method for calculating NACMEs. CH_2_NH is chosen as a test system because its excited-state properties have been reported and studied, and it has only 5 atoms [48,49,50]. Thus, the computational cost is affordable for calculating Hessian matrix elements numerically at the SA-CASSCF level.

## 4. Conclusions

In this work, we have implemented an approximate algorithm for calculating NACMEs of polyatomic systems based only on PES information, i.e., energies, gradients and Hessian matrix elements. The advantage of this algorithm is that it only demands the information of PESs without any wavefunction information, which is very suitable for machine learning techniques or low-scaling energy-based fragment methods. The approximate algorithm is used for calculating NACMEs with the ab initio SA-CASSCF method and the ML model. The results show that the PES-based algorithm for NACMEs, whether using the ab initio method or the ML model, performs very well except at the CI structure. The present work demonstrates the accuracy of the approximate PES-based algorithm for NACMEs of polyatomic systems. It encourages the combination of this approximate PES-based algorithm with electronic structure methods that do not code wavefunction-based algorithms for NACMEs. Most importantly, this PES-based algorithm provides a good opportunity to calculate NACMEs for energy-based fragment methods and ML models, in which the computational cost of Hessian matrix elements is significantly reduced, as demonstrated in our recent work, and in particular for ML models [17]. Finally, this PES-based algorithm for NACMEs in combination with either low-scaling energy-based fragment methods or efficient ML models also brings an economic but accurate nonadiabatic dynamics simulation method to investigate the nonadiabatic process of large systems.

## Figures and Tables

**Table 1:** Average norms (in Bohr^−1^) and relative deviations between wavefunction- and PES-based NACMEs. The energy gap of 0.17 kcal/mol is for the optimized CI structure. See text for discussion.

Energy Gap (kcal/mol)	Wavefunction-Based	PES-Based	Deviation
0.17 (CI)	363.4	240.9	33.7%
0.17–1	78.2	80.4	2.8%
1–3	30.3	31.4	3.6%
3–5	15.3	15.9	3.8%
5–10	8.3	8.7	4.0%
10–15	5.1	5.3	3.6%
>15	3.3	3.4	3.4%

**Table 2:** Average norms (in Bohr^−1^) and relative deviations of SA-CASSCF- and ML-calculated NACMEs (PES-based algorithm). The energy gap of 0.17 kcal/mol is for the optimized CI structure. See text for discussion.

Energy Gap (kcal/mol)	PES-Based NACMEs (SA-CASSCF)	PES-Based NACMEs (ML)	Deviation
0.17 (CI)	240.9	62.9	73.9%
0.17–1	80.4	64.1	20.2%
1–3	31.4	30.2	3.6%
3–5	15.9	14.5	9.1%
5–10	8.7	8.7	0.6%
10–15	5.3	5.4	2.3%
>15	3.4	3.5	2.7%

## Data Availability

The data that support the findings of this study are available from the corresponding author upon reasonable request.

## Supplementary Materials

The following supporting information can be downloaded at: https://www.mdpi.com/article/10.3390/molecules28104222/s1, Table S1: Number of structures for $\Delta E_{S_{0}S_{1}}\;$in different groups; Table S2: RMSD of wavefunction- and PES-based NACMEs; Table S3: RMSD of SA-CASSCF- and ML-calculated NACMEs (PES-based algorithm); Cartesian coordinates of optimized CI structure; branching space vectors.

## References

[1] Agostini F., Curchod B.F. (2019). Different Flavors of Nonadiabatic Molecular Dynamics. Wiley Interdiscip. Rev. Comput. Mol. Sci..

[2] Baer M. (2006). Beyond Born-Oppenheimer: Electronic Nonadiabatic Coupling Terms and Conical Intersections.

[3] Chu W., Zheng Q., Akimov A.V., Zhao J., Saidi W.A., Prezhdo O.V. (2020). Accurate Computation of Nonadiabatic Coupling with Projector Augmented-Wave Pseudopotentials. J. Phys. Chem. Lett..

[4] Gibson J., Penfold T.J. (2017). Nonadiabatic Coupling Reduces the Activation Energy in Thermally Activated Delayed Fluorescence. Phys. Chem. Chem. Phys..

[5] Mai S., Marquetand P., González L. (2018). Nonadiabatic Dynamics: The Sharc Approach. Wiley Interdiscip. Rev. Comput. Mol. Sci..

[6] Matsika S. (2021). Electronic Structure Methods for the Description of Nonadiabatic Effects and Conical Intersections. Chem. Rev..

[7] Ryabinkin I.G., Nagesh J., Izmaylov A.F. (2015). Fast Numerical Evaluation of Time-Derivative Nonadiabatic Couplings for Mixed Quantum–Classical Methods. J. Phys. Chem. Lett..

[8] Subotnik J.E., Alguire E.C., Ou Q., Landry B.R., Fatehi S. (2015). The Requisite Electronic Structure Theory to Describe Photoexcited Nonadiabatic Dynamics: Nonadiabatic Derivative Couplings and Diabatic Electronic Couplings. Acc. Chem. Res..

[9] Tully J.C. (2012). Perspective: Nonadiabatic Dynamics Theory. J. Chem. Phys..

[10] Wang Z., Wu C., Liu W. (2021). Nac-Tddft: Time-Dependent Density Functional Theory for Nonadiabatic Couplings. Acc. Chem. Res..

[11] Köppel H., Gronki J., Mahapatra S. (2001). Construction Scheme for Regularized Diabatic States. J. Chem. Phys..

[12] Gonon B., Perveaux A., Gatti F., Lauvergnat D., Lasorne B. (2017). On the Applicability of a Wavefunction-Free, Energy-Based Procedure for Generating First-Order Non-Adiabatic Couplings around Conical Intersections. J. Chem. Phys..

[13] Richardson J.O. (2023). Machine Learning of Double-Valued Nonadiabatic Coupling Vectors around Conical Intersections. J. Chem. Phys..

[14] An H., Baeck K.K. (2018). Practical and Reliable Approximation of Nonadiabatic Coupling Terms between Triplet Electronic States Using Only Adiabatic Potential Energies. Chem. Phys. Lett..

[15] Baeck K.K., An H. (2017). Practical Approximation of the Non-Adiabatic Coupling Terms for Same-Symmetry Interstate Crossings by Usingadiabatic Potential Energies Only. J. Chem. Phys..

[16] Westermayr J., Gastegger M., Marquetand P. (2020). Combining Schnet and Sharc: The Schnarc Machine Learning Approach for Excited-State Dynamics. J. Phys. Chem. Lett..

[17] Chen W.-K., Zhang Y.L., Jiang B., Fang W.-H., Cui G.L. (2020). Efficient Construction of Excited-State Hessian Matrices with Machine Learning Accelerated Multilayer Energy-Based Fragment Method. J. Phys. Chem. A.

[18] Zhang Y., Xia J., Jiang B. (2021). Physically Motivated Recursively Embedded Atom Neural Networks: Incorporating Local Completeness and Nonlocality. Phys. Rev. Lett..

[19] Zhang Y., Xia J., Jiang B. (2022). Reann: A Pytorch-Based End-to-End Multi-Functional Deep Neural Network Package for Molecular, Reactive, and Periodic Systems. J. Chem. Phys..

[20] Zhang Y.L., Hu C., Jiang B. (2019). Embedded Atom Neural Network Potentials: Efficient and Accurate Machine Learning with a Physically Inspired Representation. J. Phys. Chem. Lett..

[21] Chen W.-K., Fang W.-H., Cui G.L. (2019). Integrating Machine Learning with Multi-Layer Energy-Based Fragment Method for Excited States of Large Systems. J. Phys. Chem. Lett..

[22] Domcke W., Yarkony D., Köppel H. (2004). Conical Intersections: Electronic Structure, Dynamics & Spectroscopy.

[23] Werner H.J., Meyer W. (1981). Mcscf Study of the Avoided Curve Crossing of the Two Lowest 1σ+ States of LiF. J. Chem. Phys..

[24] Keith J.A., Vassilev-Galindo V., Cheng B., Chmiela S., Gastegger M., Müller K.-R., Tkatchenko A. (2021). Combining Machine Learning and Computational Chemistry for Predictive Insights into Chemical Systems. Chem. Rev..

[25] Rupp M., Tkatchenko A., Müller K.-R., von Lilienfeld O.A. (2012). Fast and Accurate Modeling of Molecular Atomization Energies with Machine Learning. Phys. Rev. Lett..

[26] Shen X., Chen J., Zhang Z., Shao K., Zhang D.H. (2015). Methane Dissociation on Ni(111): A Fifteen-Dimensional Potential Energy Surface Using Neural Network Method. J. Chem. Phys..

[27] Häse F., Valleau S., Pyzer-Knapp E., Aspuru-Guzik A. (2016). Machine Learning Exciton Dynamics. Chem. Sci..

[28] Raccuglia P., Elbert K.C., Adler P.D.F., Falk C., Wenny M.B., Mollo A., Zeller M., Friedler S.A., Schrier J., Norquist A.J. (2016). Machine-Learning-Assisted Materials Discovery Using Failed Experiments. Nature.

[29] Chmiela S., Tkatchenko A., Sauceda H.E., Poltavsky I., Schütt K.T., Müller K.-R. (2017). Machine Learning of Accurate Energy-Conserving Molecular Force Fields. Sci. Adv..

[30] Li Y., Li H., Pickard F.C., Narayanan B., Sen F.G., Chan M.K.Y., Sankaranarayanan S.K.R.S., Brooks B.R., Roux B. (2017). Machine Learning Force Field Parameters from Ab Initio Data. J. Chem. Theory Comput..

[31] Liu Q., Wang J., Du P., Hu L., Zheng X., Chen G. (2017). Improving the Performance of Long-Range-Corrected Exchange-Correlation Functional with an Embedded Neural Network. J. Phys. Chem. A.

[32] Yao K., Herr J.E., Parkhill J. (2017). The Many-Body Expansion Combined with Neural Networks. J. Chem. Phys..

[33] Dral P.O., Barbatti M. (2021). Molecular Excited States through a Machine Learning Lens. Nat. Rev. Chem..

[34] Fu B.N., Zhang D.H. (2018). Ab Initio Potential Energy Surfaces and Quantum Dynamics for Polyatomic Bimolecular Reactions. J. Chem. Theory Comput..

[35] Hu D.P., Xie Y., Li X.S., Li L.Y., Lan Z.G. (2018). Inclusion of Machine Learning Kernel Ridge Regression Potential Energy Surfaces in On-the-Fly Nonadiabatic Molecular Dynamics Simulation. J. Phys. Chem. Lett..

[36] Behler J., Parrinello M. (2007). Generalized Neural-Network Representation of High-Dimensional Potential-Energy Surfaces. Phys. Rev. Lett..

[37] Dral P.O., von Lilienfeld O.A., Thiel W. (2015). Machine Learning of Parameters for Accurate Semiempirical Quantum Chemical Calculations. J. Chem. Theory Comput..

[38] Dral P.O., Barbatti M., Thiel W. (2018). Nonadiabatic Excited-State Dynamics with Machine Learning. J. Phys. Chem. Lett..

[39] Wang H., Yang W.T. (2018). Force Field for Water Based on Neural Network. J. Phys. Chem. Lett..

[40] Wang H., Yang W.T. (2018). Toward Building Protein Force Fields by Residue-Based Systematic Molecular Fragmentation and Neural Network. J. Chem. Theory Comput..

[41] Zhang L., Han J., Wang H., Car R., Weinan E. (2018). Deep Potential Molecular Dynamics: A Scalable Model with the Accuracy of Quantum Mechanics. Phys. Rev. Lett..

[42] Liao K., Dong S., Cheng Z., Li W., Li S. (2022). Combined Fragment-Based Machine Learning Force Field with Classical Force Field and Its Application in the Nmr Calculations of Macromolecules in Solutions. Phys. Chem. Chem. Phys..

[43] Zhang Y., Lin Q., Jiang B. (2022). Atomistic Neural Network Representations for Chemical Dynamics Simulations of Molecular, Condensed Phase, and Interfacial Systems: Efficiency, Representability, and Generalization. Wiley Interdiscip. Rev. Comput. Mol. Sci..

[44] Zhang Y.-L., Zhou X.-Y., Jiang B. (2017). Accelerating the Construction of Neural Network Potential Energy Surfaces: A Fast Hybrid Training Algorithm. Chin. J. Chem. Phys..

[45] Aquilante F., Autschbach J., Baiardi A., Battaglia S., Borin V.A., Chibotaru L.F., Conti I., De Vico L., Delcey M., Galván I.F. (2020). Modern Quantum Chemistry with [Open]Molcas. J. Chem. Phys..

[46] Fdez. Galván I., Vacher M., Alavi A., Angeli C., Aquilante F., Autschbach J., Bao J.J., Bokarev S.I., Bogdanov N.A., Carlson R.K. (2019). Openmolcas: From Source Code to Insight. J. Chem. Theory Comput..

[47] Frisch M.J., Trucks G.W., Schlegel H.B., Scuseria G.E., Robb M.A., Cheeseman J.R., Scalmani G., Barone V., Petersson G.A., Nakatsuji H. (2016). Gaussian 16, Revision A. 03.

[48] Chen W.-K., Liu X.-Y., Fang W.-H., Dral P.O., Cui G.L. (2018). Deep Learning for Nonadiabatic Excited-State Dynamics. J. Phys. Chem. Lett..

[49] Frank I., Hutter J., Marx D., Parrinello M. (1998). Molecular Dynamics in Low-Spin Excited States. J. Chem. Phys..

[50] Fabiano E., Keal T.W., Thiel W. (2008). Implementation of Surface Hopping Molecular Dynamics Using Semiempirical Methods. Chem. Phys..

