# Supplementary material for: Nonadiabatic Derivative Couplings Calculated Using Information of Potential Energy Surfaces without Wavefunctions: Ab Initio and Machine Learning Implementations
Source: Molecules. 2023 May 21;28(10):4222. doi: 10.3390/molecules28104222 (PMC10223463; doi:10.3390/molecules28104222)
Supplement: Supplementary file 1 [file molecules-28-04222-s001.zip › molecules-2383339-supplementary.pdf]

Supplementary Materials: Nonadiabatic Derivative Couplings Calculated Using Information of Potential Energy Surfaces without Wavefunctions: Ab Initio and Machine Learning Implementations

Wen-Kai Chen[1,2], Sheng-Rui Wang[2], Xiang-Yang Liu[4], Wei-Hai Fang[2,3], Ganglong Cui[2,3*]

[1] College of Chemistry and Materials Science, Hebei Key Laboratory of Inorganic Nano-Materials, Hebei Normal University, Shijiazhuang 050024, China
[2] Key Laboratory of Theoretical and Computational Photochemistry, Ministry of Education, College of Chemistry, Beijing Normal University, Beijing 100875, China
[3] Hefei National Laboratory, Hefei 230088, China
[4] College of Chemistry and Material Science, Sichuan Normal University, Chengdu 610068, China

E-mail: ganglong.cui@bnu.edu.cn

1. Additional Data

Table S1. Number of structures for $\Delta E_{S_0S_1}$ in different groups.

**Energy Gap (kcal/mol)**	**Number of Data**
0.17 (MECI)	1
0.17-1	5
1-3	42
3-5	85
5-10	379
10-15	319
> 15	130

Table S2. Root mean square deviation (RMSD) values (in $Bohr^{-1}$) of the PES-based NACMEs with respect to the wavefunction-based NACMEs.

**Energy Gap (kcal/mol)**	**RMSD ($Bohr^{-1}$)**
0.17 (MECI)	86.09
0.17-1	36.71
1-3	2.24
3-5	1.15
5-10	0.66
10-15	0.41
> 15	0.27

Table S3. Root mean square deviation (RMSD) values (in $Bohr^{-1}$) of the SA-CASSCF- and ML-calculated NACMEs (PES-based algorithm).

Energy Gap (kcal/mol)	RMSD ($Bohr^{-1}$)
0.17 (MECI)	46.79
0.17-1	33.71
1-3	6.09
3-5	1.46
5-10	0.40
10-15	0.19
> 15	0.17

2. Cartesian Coordinates of the optimized CI structure (In Å)

C	0.6469	-0.0000	0.0502
H	1.1917	0.9240	-0.0041
H	1.1888	-0.9254	-0.0045
N	-0.7133	0.0002	-0.1428
H	-1.2689	-0.0002	0.7074

3. Branching space vectors at the optimized CI structure

g

-0.0547998078	-0.0289750951	-0.0267761876
0.0005912536	-0.0007005861	-0.0326824556
0.0037434127	-0.0011734331	0.0284251728
0.0631510096	0.0975623138	0.0485796303
-0.0126858681	-0.0667131995	-0.0175461599

h

-0.0619946799	0.0134129868	-0.0074055181
0.0073314251	-0.0005031829	0.0067776450
0.0029965689	-0.0022956584	-0.0123248759
0.0632778457	-0.0270384598	0.0279724305
-0.0116111599	0.0164243143	-0.0150196815
